# Mild Neonatal Brain Hypoxia-Ischemia in Very Immature Rats Causes Long-Term Behavioral and Cerebellar Abnormalities at Adulthood

**DOI:** 10.3389/fphys.2019.00634

**Published:** 2019-06-05

**Authors:** Eduardo Farias Sanches, Yohan van de Looij, Audrey Toulotte, Stéphane Vladimir Sizonenko, Hongxia Lei

**Affiliations:** ^1^Division of Child Development and Growth, Department of Pediatrics, School of Medicine, University of Geneva, Geneva, Switzerland; ^2^Laboratory for Functional and Metabolic Imaging, Ecole Polytechnique Fédérale de Lausanne, Lausanne, Switzerland; ^3^Center for Biomedical Imaging, Ecole Polytechnique Fédérale de Lausanne, Lausanne, Switzerland

**Keywords:** hypoxia-ischemia, prematurity, ^1^H magnetic resonance spectroscopy, ^1^H MRS, cerebellum, brain

## Abstract

Systemic hypoxia-ischemia (HI) often occurs during preterm birth in human. HI induces injuries to hinder brain cells mainly in the ipsilateral forebrain structures. Such HI injuries may cause lifelong disturbances in the distant regions, such as the contralateral side of the cerebellum. We aimed to evaluate behavior associated with the cerebellum, to acquire cerebellar abundant metabolic alterations using *in vivo* ^1^H magnetic resonance spectroscopy (^1^H MRS), and to determine GFAP, NeuN, and MBP protein expression in the left cerebellum, in adult rats after mild early postnatal HI on the right forebrain at day 3 (PND3). From PND45, HI animals exhibited increased locomotion in the open field while there is neither asymmetrical forelimb use nor coordination deficits in the motor tasks. Despite the fact that metabolic differences between two cerebellar hemispheres were noticeable, a global increase in glutamine of HI rats was observed and became significant in the left cerebellum compared to the sham-operated group. Furthermore, increases in glutamate, glycine, the sum of glutamate and glutamine and total choline, only occurred in the left cerebellum of HI rats. Remarkably, there were decreased expression of MBP and NeuN but no detectable reactive astrogliosis in the contralateral side of the cerebellum of HI rats. Taken together, the detected alterations observed in the left cerebellum of HI rats may reflect disequilibrium in the glutamate-glutamine cycle and a delay in the return of glutamine from astrocytes to neurons from hypoxic-ischemic origin. Our data provides *in vivo* evidence of long-term changes in the corresponding cerebellum following mild neonatal HI in very immature rats, supporting the notion that systemic HI could cause cell death in the cerebellum, a distant region from the expected injury site.

**HIGHLIGHTS:**

- Neonatal hypoxia-ischemia (HI) in very immature rats induces hyperactivity toward adulthood.
- ^1^H magnetic resonance spectroscopy detects long-term cerebellar metabolic changes in adult rats after neonatal HI at postnatal day 3.
- Substantial decreases of expression of neuronal and myelin markers in adult rats cerebellum after neonatal cortical mild HI.

## Introduction

Complications derived from premature birth account for 29% of global neonatal deaths yearly and around 3% of total disability during the lifespan (Lawn et al., 2010; Howson et al., 2013). Premature newborns have a high incidence of neonatal brain injury (Gopagondanahalli et al., 2016) linked to subcortical white and gray matter lesions, impaired structural connectivity (Volpe et al., 2011; Salmaso et al., 2014) which cause lifelong neurodevelopment disturbances (Robinson, 2005; Allin et al., 2008; Delobel-Ayoub et al., 2009; Pyhälä, 2012; Breeman et al., 2015; Hübner et al., 2015; Thomason et al., 2017).

Neonatal hypoxia-ischemia (HI) contributes to pathologies such as cerebral palsy (CP), developmental delay, attention deficit and hyperactivity disorder (ADHD) learning deficits and others (Fatemi et al., 2009; Volpe, 2009a; Phillips et al., 2013). The most used experimental model of neonatal HI (Levine, 1960; Rice et al., 1981) consists of unilateral carotid ligation followed by a period of hypoxic exposure leading to deficits in motor coordination (Lubics et al., 2005), anxiety-related behavior and cognitive impairment in early and late development, due to lesions in hippocampus, striatum and cortex (Arteni et al., 2010; Sanches et al., 2015). Also, studies using HI at postnatal day 7 have shown that cell death occurs in brain regions that are not directly affected by ischemia, such as the cerebellum (Joyal et al., 1996; Kim et al., 2004; Northington et al., 2011) suggesting that neuronal connectivity may play a role in neurodegeneration following HI to the immature brain. The HI model performed at postnatal day 3 mimics the lesion observed in very preterm infants’ brains (Sizonenko et al., 2003; Sanches et al., 2013; Ginet et al., 2016). HI in the very immature rat brain causes disruption in cell metabolism, development and in cortical cytoarchitecture (Sizonenko et al., 2008; van de Looij et al., 2011; Misumi et al., 2016), alters the myelination pattern and leads to behavioral impairments (Huang et al., 2009; Sanches et al., 2015; Misumi et al., 2016). HI injury characteristics can be detected in infants born preterm via magnetic resonance imaging. Alderliesten et al. (2013) found high correlation between neuropathological evidence of cerebellar injury and MRI analysis (Alderliesten et al., 2013). Since cerebellum has a major role in high order brain functions, lesions in its connections with cortical and sub-cortical centers could lead not only to motor and verbal impairments (Marr, 1969; Barradas et al., 2016) but also to cognitive, affective and social disturbances (Schmahmann et al., 2008; Limperopoulos et al., 2009; Kitai et al., 2015). Strikingly, pathologic evidence of cerebellar injury in neonates has gained valuable input with the advances in numerous magnetic resonance imaging (MRI) techniques (reviewed by Smyser et al., 2018) in which many HI injury characteristics (Schneider et al., 2009; Matsufuji et al., 2017) and other early-life cerebellar impairments associated with brain injury in premature infants can be detected (Limperopoulos, 2005a,b). Despite the improving imaging techniques, early diagnosis before the formation of MRI-detectable lesions remains challenging (Gopagondanahalli et al., 2016). In addition, ^1^H MR spectroscopy (^1^H MRS) offers abundant cerebral metabolites and are applicable to neonatal HI in preterm newborns (Cheong et al., 2006; Xu and Vigneron, 2010) but remains less explored in the cerebellum and even less so in the long-term perspective. Besides, ^1^H MRS shows early alterations in brain structure and metabolism highly correlated to HI in clinical and preclinical settings (Roelants-Van Rijn et al., 2001; van de Looij et al., 2015; Xu et al., 2015) and could be used as a biomarker for late-term neurodevelopmental outcomes following HI.

The cerebellum is not classically considered a brain region vulnerable to hypoxic-ischemic insults mainly due to its relative distance from the injury site in initial phases of injury (only suffering from systemic hypoxia). However, recent data suggests the presence of cerebellar injury following experimental HI (Taylor et al., 2006; Biran et al., 2011). Since neonatal HI in very immature rats is highly variable and affects the cerebellum up to weeks later (Biran et al., 2011), and may not be detected by standard MRI in adulthood, we aimed to evaluate the long-term effects of mild HI (Sanches et al., 2018) on (1) cerebellar metabolism at adulthood using ^1^H MRS; (2) locomotor function, and (3) expression of astrocytes, neurons and myelin proteins.

## Materials and Methods

### Animals

Geneva State Animal Ethics Committee and the Swiss Federal Veterinary Service approved this study under GE/132/15 license. The experiments were performed at the EPFL (Centre d’Imagerie BioMédicale – CIBM) and CMU (UNIGE). Male and female Wistar rats were ordered from Charles River Laboratories (L’Arbresle, France). Animals were housed under standard animal facility conditions (12-h-light, 12-h-dark cycle and room temperature at 22 ± 1°C).

### Neonatal Hypoxia-Ischemia

At postnatal day 1 (PND1), the animals were counted, and the litters were culled to have between 8 and 12 animals (males and females were used in the study) to avoid differences regarding animal weights. At PND3, pups were submitted to a mild to a mild hypoxic-ischemic injury as previously described (Sizonenko et al., 2008; van de Looij et al., 2011; Sanches et al., 2018). Briefly, under isoflurane anesthesia (4% induction and 1.5–2.0% maintenance), the right carotid artery was isolated from the vagus nerve and surrounding tissue and permanently occluded with 6.0 silk thread. The surgical access was closed with Histoacryl^TM^ and Steri-strip^TM^. After a 30 min recovery period in a chamber at 37°C in room air, the flux of room air was replaced by 2 L/min of 6% O_2_ at 37°C during 30 min to induce hypoxia. Sham-operated (SH) animals were anesthetized, had the incision without carotid occlusion or hypoxia.

### Behavioral Analysis

At young adult/adolescence age (from PND45) animals performed locomotor tests. The same investigator performed all experimental sessions in a light and sound controlled room. The number of animals used for the behavioral analysis was SH = 13 and HI = 19.

#### Open Field (OF)

The test allows the observation of exploratory activity of animals in a novel environment. OF consisted of a circular wooden chamber (100 cm diameter × 30 cm high wall) with a floor divided into 21 fields. Using ANY-Maze software, the open field test was video recorded during 5 min. The latency to leave the central circle, number of crossings and rearings were considered as indicative of spontaneous motor activity (Sanches et al., 2017).

#### Cylinder Test (CYL)

This test is used to assess the asymmetrical use of forelimbs after hypoxia-ischemia (Grow et al., 2003). Animals were placed inside a Plexiglas cylinder (20 cm diameter × 40 cm high) and videotaped from the top. Spontaneous ipsilateral and contralateral forelimb wall contacts on the cylinder wall were recorded for 4 min. When the number of total contacts was less or equal to twelve, the animal was removed from the statistical analysis. The equation: (ipsilateral contacts/ipsilateral + contralateral) × 100 was used to calculate percentage of asymmetrical use of the forelimbs (Sanches et al., 2013).

#### Beam Balance (BB)

To access locomotor deficits, we modified the protocol described by Lotan et al. (2014). The rats were trained (three trials) to traverse a narrow wooden beam (width 2.5 cm, length 100 cm). The beam rested on two acrylic boxes at 50 cm above the floor. The animals were placed on one side, with a safe place (a black box) on the other side, allowing the animals to walk on the beam. In the test session, the number of hindpaw slips were counted (in three trials) 24 h after the training session.

### *In vivo* ^1^H Magnetic Resonance Spectroscopy (^1^H MRS)

Following behavioral analysis, ^1^H MRS was carried out in a horizontal, 14.1-T/26-cm magnet (Magnex Scientific, United Kingdom), equipped with a 12-cm inner-diameter gradient (400 mT/m in 200 μs, minimized eddy currents) and interfaced with a DirectDrive console (Varian Inc., Palo Alto, CA, United States). A home-built quadrature surface coil with two geometrically decoupled single-turn loops (16-mm inner diameter), resonating at 600 MHz radio frequency (RF), was used as RF transceiver. Briefly, as previously described (Lei et al., 2009), animals 15 females (7 SH and 8 HI) and 10 males (3 SH and 7 HI) were anesthetized with 5% isoflurane mixed in O_2_ and air (1:1) and then maintained under 1.5–2.5% isoflurane during the entire of MR session (∼50–55 min). Once animal heads were stereotaxically fixed by two ear pieces and one bite bar, animals were secured into a home-built holder and transferred to the center of the magnet. During the entire experiment, the animals were monitored for breathing rates (∼60 breaths-per-min) and rectal temperature (∼37°C) through a MR-compatible monitor system (Model 1025, SA Instruments Inc., Stony Brook, NY, United States).

Multislice T_2_-weighted images were acquired using the fast spin-echo technique [FSE (Hennig, 1988)], with effective echo time TE_eff_ = 50 ms, repetition time TR = 4000 ms and 4 averages (∼6 min). Thereafter, both first- and second-order shim terms over the VOI were altered accordingly using FASTMAP (Gruetter and Tkáč, 2000) and resulted in water linewidth <20 Hz for a 15 μL volume. Localized ^1^H-MR spectra of both cerebellar hemispheres were obtained using the SPECIAL technique (Mlynárik et al., 2006), TE/TR = 2.8/4000 ms and 240 averages (16 min) in combination with outer volume suppression and VAPOR water suppression. The corresponding non-water suppressed spectra (eight averages) were acquired for further quantification (assuming 80% water in both hemispheres) of the cerebellum (McBride et al., 2018).

In this study, metabolites were processed and analyzed using the LCModel (Lei et al., 2009 and references therein). In particular, acetate (Ace), alanine (Ala), ascorbate (Asc), aspartate (Asp), creatine (Cr), myo-inositol (Ins), γ-aminobutyric acid (GABA), glutamine (Gln), glutamate (Glu), glycine (Gly), glycerophocholine (GPC), glutathione (GSH), lactate (Lac), *N*-acetyl-aspartate (NAA), *N*-acetyl-aspartyl-glutamate (NAAG), phosphocholine (PCho), phosphocreatine (PCr) phosphoethanolamine (PE), scyllo-inositol (scyllo), macromolecules (Mac), and taurine (Tau) were quantified. Summed concentrations, e.g., Glu+Gln, PCr+Cr, GPC+PCho, and NAA+NAAG, were also calculated. Based on our preliminary data, Scyllo and Ace were noticeably less than 0.2 μmol/g among all spectra of both groups and considered to be non-detectable. No cerebellar volumetric data was analyzed in the study.

### Immunoblotting

Total RNA and proteins were extracted with PrepEase RNA/Protein Spin Kit (78871 1 KT; Affymetrix, Santa Clara, CA, United States) according to the manufacturer’s instructions. Protein pellets were resuspended in RIPA buffer (Cell Signaling, 9806S). For immunoblotting, protein extracts were sonicated and the protein concentration was determined using a Bradford assay. Proteins (25 μg) were separated by SDS-PAGE, transferred to nitrocellulose membrane and analyzed, as previously described (Sanches et al., 2018). Briefly, after overnight incubation with primary antibodies for neurons (NeuN, Sigma-Aldrich), astrocytes (GFAP, Sigma-Aldrich) and myelin (MBP, Abcam) were diluted (1:1000) in 0.1% casein (Sigma-Aldrich, C8654) membranes were incubated with the following secondary antibodies (1:10000): goat anti-mouse IgG conjugated with IRDye 680 (LI-COR, B70920-02), goat anti-rabbit IgG conjugated with IRDye 800 (LI-COR, 926-32210). Protein bands were visualized using the Odyssey Infrared Imaging System (LI-COR). ImageStudio^TM^ Lite (LI-COR) was used to measure the optical densities of protein signals on all scans. The optical density of each sample was first estimated based on the optical density of a loading control (βIII-tubulin), and then normalized to the corresponding SH value (as 100%) (*n* = 6–8 animals/group). Original western blotting images are presented in the Data Sheet S1 (clearly marked in Supplementary Figure S1) and the list of antibodies used in the study are presented in the Supplementary Table S1. RNA was not analyzed in the study.

### Statistical Analysis

All statistical analyses were performed using SPSS 19.0 for Windows (SPSS Inc., Chicago, IL, United States). Data are presented as mean ± standard error of the mean (SEM). All comparisons between SH and HI groups were made using unpaired *t*-tests. For the comparisons between hemispheres, paired t–tests were performed. Two-way ANOVA (GraphPad Prism) was carried out for analyzing treatment factor (HI vs. SH) and one additional factor, e.g., behavior, metabolite and protein expression, respectively. The significance was accepted when *p* < 0.05.

## Results

### Behavioral Analysis

Due to the role of the cerebellum in locomotion, motor function was evaluated from PND45 (Table 1). In the OF test, HI animals presented hyperactivity, i.e., increased number of crossings (*t* = -2.357, *p* = 0.026), compared to their controls. Other parameters evaluated in the OF test, such as latency to leave the center of the arena and the number of rearings, did not show significant differences between groups. Furthermore, there is no statistically significant differences in either the beam balance number of mistakes in the paw placements (*t* = -0.658, *p* = 0.515) or asymmetrical use of the forelimbs in the cylinder test (*t* = 0.025, *p* = 0.77). Taken together, two-way ANOVA on factors of treatment and behavioral outcomes revealed substantial differences in the treatment factor (*p* = 0.01).

**Table 1:** Summary of behavioral analysis in the open field, beam balance errors, and asymmetrical use of forelimbs in the Cylinder test.

		SHAM (*n* = 13)	HI^(†)^ (*n* = 19)
Open field (OF) – Latency to leave the center		1.4 ± 0.3	2.1 ± 0.5
	Number of crossings	179 ± 11.3	207 ± 5.7^∗^
	Number of rearings	15.5 ± 2.7	18.5 ± 2.3
Cylinder (%) – asymmetrical use of the forelimbs		52.4 ± 1.7	51.4 ± 1.4
Beam balance – number of errors		1.3 ± 0.1	1.6 ± 0.3


*Data are expressed as mean ± SEM. The results were analyzed by unpaired *t*-test between HI and SHAM. Significance was accepted when *p* < 0.05, as marked “^∗^”. Two-way ANOVA of treatment and behavioral test factors resulted in significant difference in treatment (*p* = 0.01, “^†^”).*

### Cerebellar Metabolism

In order to search for clinically relevant information on long-term cerebellar metabolism alterations following neonatal HI at PND3 and evaluating potential *in vivo* biomarkers, non-invasive ^1^H MRS was performed on both cerebella of neonatal HI and SH rats from PND50.

**FIGURE 1:**
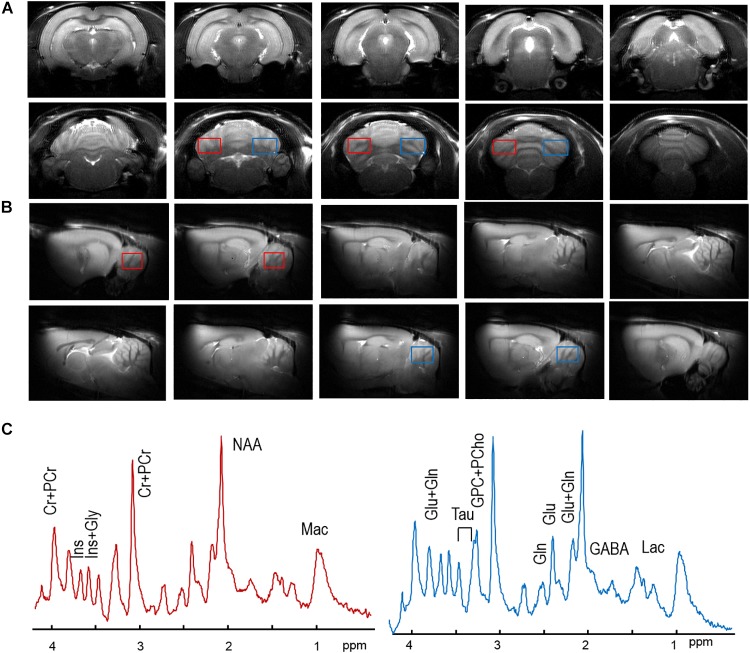
Typical axial **(A)** and sagital **(B)** MR images, and one of each spectrum from the right cerebellum (red boxes) and the left cerebellum (blue boxes) in and **(B)** of one SH rat. In panel **(C)**, no visual difference was observed between the right cerebellar spectrum (in solid red line) and the left one (in solid blue line). No visual differences in MR spectra were observed. Major metabolic resonances were highlighted along with their abbreviations, as listed in the Section “Materials and Methods.”

**FIGURE 2:**
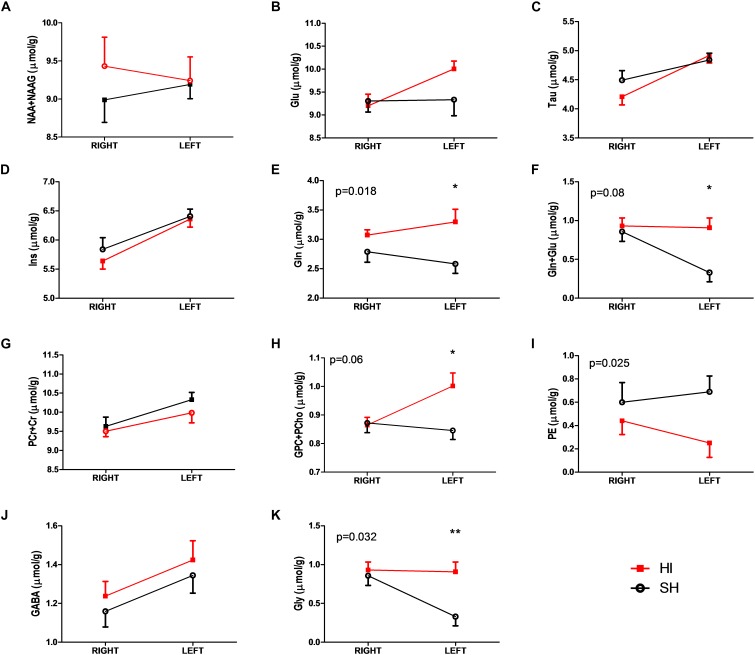
Selected metabolic differences **(A–K)** between SH (open black circles) and HI (solid red squares) groups of adult rats following HI and SH at PND3. Two-way ANOVA analysis showed treatment differences with the corresponding *p*-values **(E,J,K)**. Total choline (GPC+PCho, **H**) and total glutamine and glutamate (Gln+Glu, **F**) showed different trends toward the significant level, i.e., *p* < 0.05. The further Bonferroni post-test revealed further differences, *p*-value <0.05 was marked with “^∗^” and *p*-value <0.01 was marked with “^∗∗^.” Abbreviations were listed in the “Materials and Methods.”

High quality anatomical images did not show brain abnormalities or T_2_-hyperintensities in either cerebral cortex or cerebellum in HI rats compared to the SH group. The quality of the images allowed precise location of both volumes of interests, i.e., right and left hemispheres of cerebellum (Figure 1A,B). Spectral quality was evaluated based on the improvements of field homogeneities, efficiency of water suppression and sufficient signal-to-noise ratios (SNRs) by accumulation of scans, as explained in methods. For instance, in SH animals, the resulting metabolic linewidth of right hemisphere was 14.4 ± 0.9 Hz and that of left hemisphere was 12.6 ± 0.9 Hz after the improvement of field homogeneities. With averaging the sufficient number of scans (i.e., 240), SNRs of 14.9 ± 0.7 and 14.4 ± 0.7 were achieved in the spectra of the right and left hemispheres (Figure 1C), respectively. Such spectral data allowed up to 20 metabolites to be reliably quantified (Figure 2 and Table 2).

**Table 2:** Summary of additional cerebellar metabolic results obtained from localized ^1^H-MRS.

		Right (SH vs. HI)	Left (SH vs. HI)	Right hemisphere	Right vs. left (paired *t*-test)	Left hemisphere
**Mac**	SH (*n* = 9)			1.38 ± 0.01		1.41 ± 0.04
	HI (*n* = 15)			1.33 ± 0.04		1.40 ± 0.05
**Ala**	SH			0.39 ± 0.09		0.39 ± 0.08
	HI			0.41 ± 0.08		0.39 ± 0.06
**Asp**	SH			1.12 ± 0.2		0.78 ± 0.20
	HI			1.25 ± 0.2	*p* = 0.09	0.85 ± 0.14
**PCho**	SH			0.63 ± 0.11		0.60 ± 0.12
	HI			0.66 ± 0.08		0.69 ± 0.07
**Cr**	SH	*p* = 0.08		5.24 ± 0.10	*p* = 0.002	5.77 ± 0.16
	HI			5.59 ± 0.16	*p* = 0.06	6.10 ± 0.17
**PCr**	SH			4.26 ± 0.18		4.21 ± 0.15
	HI			4.04 ± 0.12		4.22 ± 0.17
**GABA**	SH			1.16 ± 0.08	*p* = 0.055	1.34 ± 0.09
	HI			1.24 ± 0.08		1.42 ± 0.10
**Lac**	SH			0.87 ± 0.09		0.94 ± 0.10
	HI			0.67 ± 0.10		0.77 ± 0.08
**NAA**	SH			8.50 ± 0.34		8.05 ± 0.32
	HI			8.08 ± 0.29		8.20 ± 0.20
**Asc**	SH			0.90 ± 0.21	*p* = 0.057	1.55 ± 0.30
	HI			0.94 ± 0.17		1.03 ± 0.18
**Glc**	SH			2.41 ± 0.48		1.87 ± 0.22
	HI			1.92 ± 0.24		1.81 ± 0.30
**GPC**	SH			0.24 ± 0.11		0.24 ± 0.11
	HI			0.20 ± 0.08		0.32 ± 0.08


*All data were shown as mean ± SEM. Abbreviations were listed in methods. Paired *t*-test was performed in the same animals. Unpaired *t*-test was performed between groups, i.e., SH vs. HI.*

Paired *t*-tests showed that most metabolites in the SH group were similar between right and left hemispheres of the cerebellum, except for Cr (5.2 ± 0.1 vs. 5.8 ± 0.2 μmol/g, *p* = 0.002), GSH (0.7 ± 0.1 vs. 0.4 ± 0.1 μmol/g, *p* = 0.027), Gly (0.9 ± 0.1 vs. 0.3 ± 0.1 μmol/g, *p* = 0.003), and Ins (5.8 ± 0.2 vs. 6.4 ± 0.1 μmol/g, *p* = 0.037).

In the HI group, metabolic differences between right and left hemispheres of cerebella were observed: e.g., Glu (9.2 ± 0.3 vs. 10.0 ± 0.2 μmol/g, *p* = 0.008), Ins (5.6 ± 0.1 vs. 6.4 ± 0.1 μmol/g, *p* = 0.001), Tau (4.2 ± 0.1 vs. 4.9 ± 0.1 μmol/g, *p* = 0.001), Glu+Gln (12.3 ± 0.3 vs. 13.1 ± 0.2 μmol/g, *p* = 0.034), GPC+PCho (0.9 ± 0.03 vs. 1.0 ± 0.05 μmol/g, *p* = 0.007), and Cr+PCr (10.3 ± 0.2 vs. 9.6 ± 0.2 μmol/g, *p* = 0.03). Interestingly, the observed differences of GSH and Gly between hemispheres in the SH group were abolished (*p* ≥ 0.8) in the HI group (Figure 2).

Unpaired *t*-tests revealed noticeable metabolic changes in the left cerebellum two months after neonatal HI compared to the corresponding SH values: increases in the concentrations of Gln (HI vs. SH: 3.1 ± 0.1 vs. 2.6 ± 0.2 μmol/g, *p* = 0.02), Gly (0.9 ± 0.1 vs. 0.3 ± 0.1 μmol/g, *p* = 0.003), Glu+Gln (13.1 ± 0.2 vs. 11.9 ± 0.4 μmol/g, *p* = 0.034), and GPC+PCho (1.0 ± 0.5 vs. 0.8 ± 0.03 μmol/g, *p* = 0.01) along with decrease in PE (0.2 ± 0.1 vs. 0.7 ± 0.2 μmol/g, *p* = 0.027) (Figure 2). Additional alteration trends were noticeable in Glu (10.0 ± 0.2 vs. 9.3 ± 0.4 μmol/g, *p* = 0.11) and NAAG (1.0 ± 0.1 vs. 1.2 ± 0.1 μmol/g, *p* = 0.053) in the left cerebellum of HI animals compared to their respective SH group. However, all metabolites did not show significant differences between right cerebellum hemispheres in both HI and SH groups (Figure 2 and Table 2). Further two-way ANOVA confirmed HI-induced differences in GPC+PCho (*p* = 0.06) and even more so in Gln (*p* = 0.018) and PE (*p* = 0.025), as shown in Figure 2.

### Cell Markers Protein Expression

**FIGURE 3:**
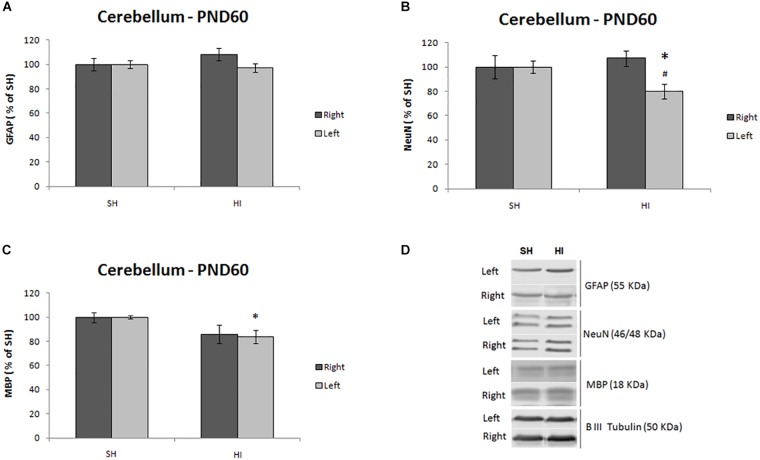
Summary of protein expression of astrocytes (GFAP, **A**), neurons (NeuN, **B**), and myelin (MBP, **C**) in both hemispheres of the cerebellum in adult rats (PND60) after HI or SH at PND3. The representative blots for the NeuN, GFAP, and MBP, for SH and HI groups, were shown in panel **(D)**. Western blotting results are plotted normalized to SH expression level (100%) (mean ± SEM). “^∗^” Stands for difference between HI and SH (*n* = 6–8 animals per group), and “^#^” represents difference between right and left hemispheres. Significance was determined using unpaired *t*-test when *p* < 0.05.

Immediately after ^1^H MRS, protein expression analysis was performed in both cerebellum hemispheres to evaluate plausible anomalies in the expression of proteins related to neurons, myelin and astrocytes. A significant decrease in the expression of NeuN (*t* = 2.59, *p* = 0.029) was observed in the left cerebellum of HI animals (Figure 3B,C) compared to SH group. Differences between right and left cerebellar hemispheres were observed in the HI group for NeuN only (*t* = 3.492, *p* = 0.006) (Figure 3B). Similarly, hypomyelination was observed through the decrease in MBP expression in the contralateral left cerebellar hemisphere of HI animals compared to SH rats (*t* = 2.381, *p* = 0.027) (Figure 3B). Interestingly, despite the decrease in MBP expression in the right hemisphere, no significant differences were observed. Despite metabolic alterations observed in ^1^H MRS, reactive astrogliosis was not significantly altered between groups; however, a trend to an increase in GFAP in the right hemisphere was observed in the HI group (*t* = 2.322, *p* = 0.053) (Figure 3A).

## Discussion

To our knowledge, this study is the first to report long-lasting effects of mild neonatal HI on cerebellar metabolism with extension to neuropathology of adult rats. Taking together behavior and motor performances, our *in vivo* ^1^H MRS findings suggest that neonatal HI at PND3 has consequences on cerebellar metabolism and function of rats in adulthood. Furthermore, in the targeted cerebellum of HI rats, protein expression of both neuronal and myelin markers was reduced compared to their respective controls despite no evidence of damage. Collectively, our study provides relevant *in vivo* evidences that neonatal HI, one of the main causes of Periventricular Leukomalacia and Cerebral Palsy, affects brain pathology in adulthood and induces alterations distant from the injury site, such as in the cerebellum.

### Neonatal HI Induces Long-Term Cerebellar Metabolic Alterations in ^1^H MRS

With the aim of investigating potential effects of neonatal HI on abundant metabolites in the cerebellum, we studied both hemispheres in adult rats after neonatal HI and SH using non-invasive ^1^H MRS.

### Primary Energy Disturbances Right After Neonatal HI Are Restored in Adulthood

Following HI, there is a primary phase of energy failure up to 24 h following injury, with a decrease in energetic brain metabolites such as ATP and PCr (Blumberg et al., 1996; van de Looij et al., 2011; Thornton and Hagberg, 2014). Given the intensity and the initial cortical target injury following neonatal HI (i.e., 30 min hypoxia exposure) and the age at time of analysis of rats in this study, energy related substrates should return to normal levels (van de Looij et al., 2011). In particular, lactate in the targeted cerebellum of HI rats was not different from SH animals. In addition, PCr in the targeted cerebellar hemisphere of HI rats (4.2 μmol/g) was identical to the SH rats (4.2 μmol/g), while a slight increase in Cr in the left cerebellum of HI rats was observed. Thus, the normal levels of energy related substrates in adult rats after neonatal HI at PND3 were restored following the initial phase of energy failure immediately after neonatal HI, e.g., drop in PCr and increase in Lac (van de Looij et al., 2011).

### Hemispherical Differences in Cerebellar Metabolites Altered by Neonatal HI

Although selected metabolites were noticeably different between the two hemispheres of the cerebellum (Figure 2 and Table 2), substantial metabolic changes in the targeted cerebellum of HI rats remained observable when compared to their respective SH (Figure 2 and Table 2). It is also interesting to note that some hemisphere differences occurring in the SH animals disappeared in HI rats, e.g., GSH and Gly. Although we could not exclude the fact that Gly is highly overlapped with one Ins resonance in the typical ^1^H MR spectrum, the very similar quality of spectral data (SNR >10, metabolic linewidth ∼14 Hz) and another non-overlapping resonance of Ins provides reliable quantification of both Gly and Ins at 14.1T (Gambarota et al., 2008; Mlynárik et al., 2008). Thus, SH discrepancies could be largely due to some inheriting technical differences, e.g., coil sensitivity or chemical shift error (Mlynárik et al., 2006).

### Astrocytic and Neuronal Specific Metabolic Pool Alterations

Myo-inositol (Ins), a glial specific metabolite, exhibited hemispherical differences but was not significantly different between groups in our study (Figure 2). In addition, the concentration of putative neuronal markers, NAA and its downstream product (NAAG), did not level off from their control values (Table 2). Altogether, this suggests an absence of neuronal death or suffering as well as glial reaction 2 months after HI. Furthermore, while Glu was not reduced in the left hemisphere of HI cerebellum, Gln in both cerebellar hemispheres of the HI rats were elevated compared to their control levels, even more so in the left hemisphere (*p* < 0.05). Since Gln is mainly located in astrocytes, this elevation in Gln may be associated with astrocytic uptake of excessive extracellular glutamate, which has been shown to occur shortly after transient ischemia (Lei et al., 2009).

### Membrane Phospholipid Changes in the Cerebellum

It is noteworthy that PE decreases in the cerebellum of adult rats after neonatal HI (Figure 2). PE is a precursor to one of the major constituents of the phospholipid bilayer of cellular membranes, phosphatidylethanolamine, which restricts primarily in the inner leaflet. Since PE decreases with brain development in rodents paralleling the progression of myelination and cell proliferation (Gyulai et al., 1984; Blüml et al., 1999; Tkáč et al., 2003), the lower cerebellar PE here may not be fully explained by delayed brain development, given the lower expression of neurons and myelin markers observed in our study (Figure 3).

In addition, the sum of two other choline containing compounds of membrane lipid synthesis, i.e., GPC+PCho, is noticeably elevated in the left hemisphere of HI rats (Figure 2 and Table 2). Since PE is the substrate of membrane synthesis and GPC is one membrane breakdown product, our ^1^H MRS data suggests that membrane synthesis is likely incapable of compensating membrane breakdown in the cerebellum of HI rats. Further reduction of PCho/GPC (∼2.2) in the left cerebellum of HI rats compared to that of SH rats (∼2.5) reinforces such a notion, indicating a possible decrease in cell turnover. Together, *in vivo* ^1^H MRS of PE is in agreement with the expression of myelin basic protein (MBP) marker.

### Glutamatergic Neurotransmission Is Altered in the Cerebellum of Adult Rats After Neonatal HI

Cerebral glutamine is synthesized from extracellular Glu and/or ammonia by glutamine synthase (GS) in the astrocytes. Given that the ammonia present in the blood stream has been detoxified by the liver, it has been postulated that the elevated Gln levels upregulate uptake of extracellular glutamate, especially upon the restoration of reperfusion after acute stroke (Lei et al., 2009). Glu, on the other hand, is mainly located in the neurons and its concentration increase may be associated with numerous factors, namely extracellular glutamate accumulation from cerebellar and/or other efferent neurons, reduction of glutamate transporter and increase in glutamatergic neuron population.

In the absence of significant GFAP changes (Figure 3A), elevated concentrations of Gln in the cerebellum of adult rats after neonatal HI doesn’t seem to be associated with astrogliosis. Instead, such Gln increase may be related to the uptake of extracellular glutamate into astrocytes. In addition, the reduction of protein expression of NeuN did not fully support the notion that accumulation of Glu may be due to the increase in glutamatergic neuron population. Although we could not exclude either some loss of glutamate transporters at this stage (Torp et al., 1995; Raymond et al., 2011) or the reduction of NeuN in specific cells, e.g., Purkinje cells (Biran et al., 2011), and given the role of the cerebellum in the motor control system (Volpe, 2009b), the accumulation of Glu might partially occur due to the afferent and efferent trans-synaptic connections between cerebellum and other brain regions, e.g., motor cortex and brainstem, respectively.

Given the vulnerability of the cerebellum (Volpe, 2009b), cerebellar damage may be independent of the extend of forebrain injury (Biran et al., 2011). Thus, we hypothesize that the cerebellum after mild neonatal HI (Sizonenko et al., 2008; Sanches et al., 2018) continues to be affected for prolonged periods into adulthood. As no protein overexpression of either astrocytes or neurons was observed, and that the enlarged metabolic pool sizes of glutamatergic neurotransmission with no reduction in either GABAergic or glycinergic pools occurred in the cerebellum even in adulthood following the very mild neonatal HI injury (Figure 2), our data suggests that glutamatergic neurotransmission in the left cerebellum of rats (after right forebrain HI) is altered mostly due to trans-synaptic connections.

Although neurogenesis is nearly complete after birth, Purkinje cells start elaborating their characteristic of dendritic arbors up to PND5 (Sotelo, 2004). In the present study, the protein expression of NeuN was noticeably decreased. This observation is in line with the aforementioned Purkinje cell loss weeks after HI at PND2 and further supported by increased number of apoptotic cells in the internal granular layer day(s) after neonatal HI at PND7 and PND14 (Peng et al., 2005; Taylor et al., 2006).

Altogether, these results indicate that HI injury to the right forebrain, irrespective of the underlying mechanism, induces cell loss in the left cerebellar hemisphere, a brain region not experiencing hypoxic-ischemic insult (Vannucci et al., 1988) and distant from the primary injury.

### Metabolic Alterations May Be Associated With Hyperactivity

Premature birth impedes cerebellar development even in the absence of detectable brain injury (Limperopoulos, 2005b). Children suffering from perinatal HI exhibit neurological disorders, learning disabilities, hyperactivity, visual impairments and other limitations that compromise their life quality (Aarnoudse-Moens et al., 2009; Volpe, 2009a). In particular, preterm babies suffering unilateral cerebral injuries show cerebellar damage in the contralateral hemisphere (Limperopoulos, 2005a). Similarly, in rats following neonatal HI at PND7, mild to marked locomotor abnormalities were reported, including shorter intervals for falling from rotarod, impairments in beam walking (McQuillen and Ferriero, 2004) and delayed motor abilities (Lubics et al., 2005). Despite being less investigated than HI at PND7 (Ikeda et al., 2001; McQuillen and Ferriero, 2004; Lubics et al., 2005; Lu et al., 2014), studies using HI at PND3 have shown delays in neurological reflex maturation that lead to motor deficits weeks after the insult near adulthood (Misumi et al., 2016; Durán-Carabali et al., 2017; Sanches et al., 2017). Regardless the influence of immaturity on HI brain damages (Rice et al., 1981; Towfighi et al., 1997), motor dysfunctions remain largely dependent on the severity of injury (Ikeda et al., 2001; McQuillen and Ferriero, 2004; Lubics et al., 2005; Lu et al., 2014; Misumi et al., 2016; Durán-Carabali et al., 2017). However, to date, no *in vivo* ^1^H MRS study has investigated adulthood cerebellum consequences of HI in the very immature rat.

Thus, non-invasive methodologies that enable identification of ischemic core and penumbra, while seeking plausible biomarkers and therapeutic targets, and longitudinal follow-up treatment in the very same subjects would intrinsically improve diagnosis and prognosis of neonatal HI. Here, we incorporated some well-established behavior and motor function tests (Aarnoudse-Moens et al., 2009) in adult rats after mild neonatal HI, i.e., 30 min hypoxia at PND3 (Sanches et al., 2018) in addition to abundant metabolic information that provides non-invasive biomarkers (Lei et al., 2009; Berthet et al., 2011, 2014). In the present study, after mild neonatal HI, rats showed behavior hyperactivity accompanied by very mild motor dysfunction at adult age (Table 1). Indeed, deficits in motor coordination and locomotion tasks associated with cortical damage have been reported shortly after neonatal HI (Ten et al., 2003), e.g., a disorganization of oligodendrocyte development in the sensorimotor cortex (Misumi et al., 2016).

These alterations in cerebellar metabolites (Figure 2) accompanied by cellular abnormalities (Figure 3), hyperactivity and mild motor dysfunction observed in HI rats at adulthood could be associated with (1) a disturbance in neuron-glial interaction due to myelin loss that was evidenced by altered membrane phospholipids (Figure 2) and confirmed by reduced myelination (Figure 3C); and (2) an imbalance in glutamatergic neurotransmission with increases in both glutamine and glutamate (Figure 2). In parallel, neither substantial cellular changes in the neonatal HI injured cortex (Sanches et al., 2018) nor ^1^H-MRS-detectable metabolic changes (Supplementary Figure S2) were found at this age, supporting the notion that myelin loss may change conduction velocity in trans-synaptic connections, and any remaining alterations resulting from the forebrain HI-insult may contribute toward other interneuron networks. Furthermore, our results indicate that the neuronal loss and hypomyelination due to the combination between hypoxia and ischemia was mainly observed in the left hemisphere of HI group, which confirms the unilateral injury profile, since both hemispheres are affected by systemic hypoxia.

## Conclusion

Although the precise neuropathologic characteristics, involved in the long-term damage observed in remote areas from injury site in the developing brain following mild neonatal HI, remain to be further explored, our results provide insights into the long-term cerebellar abnormalities in metabolism, cellular damage and functional alterations after mild HI in the very immature rat. Therefore, the capability of ^1^H MRS in providing useful diagnostic biomarkers after stroke and other diseases in combination with behavior and motor performance tests may improve diagnosis and prognosis in neonatal HI.

## Ethics Statement

Geneva State Animal Ethics Committee and the Swiss Federal Veterinary Service approved this study under GE/132/15 license.

## Author Contributions

ES, SS, and HL designed the study. ES performed the HI, behavioral analysis, and WB quantification. HL performed the ^1^H MRS experiments and analyzed the data. ES and AT performed the WB. ES and HL wrote the manuscript. ES, YvdL, SZ, and HL revised the manuscript.

## Conflict of Interest Statement

The authors declare that the research was conducted in the absence of any commercial or financial relationships that could be construed as a potential conflict of interest.

## Supplementary Material

FIGURE S1 Original western blotting runs. Note that only the 7 ladders localized in the left side of the gels were used in the study (*n* = 6–8 animals/group). Antibodies used in the study were NeuN **(a)**, GFAP **(b)**, βIII Tubulin **(c)**, and MBP **(d)**.

FIGURE S2 Metabolite concentrations in the brain cortex (R: right and L: left) after neonatal HI compared to the sham-operated animals (SH). Metabolite abbreviations were presented in the Methods section. No substantial differences were observed.

TABLE S1 Antibodies used in the study.

DATA SHEET S1 The raw data of typical western blotting runs.
