# Supplementary figures and images for: Mild Neonatal Brain Hypoxia-Ischemia in Very Immature Rats Causes Long-Term Behavioral and Cerebellar Abnormalities at Adulthood
Source: Front Physiol. 2019 Jun 5;10:634. doi: 10.3389/fphys.2019.00634 (PMC6560160; doi:10.3389/fphys.2019.00634)

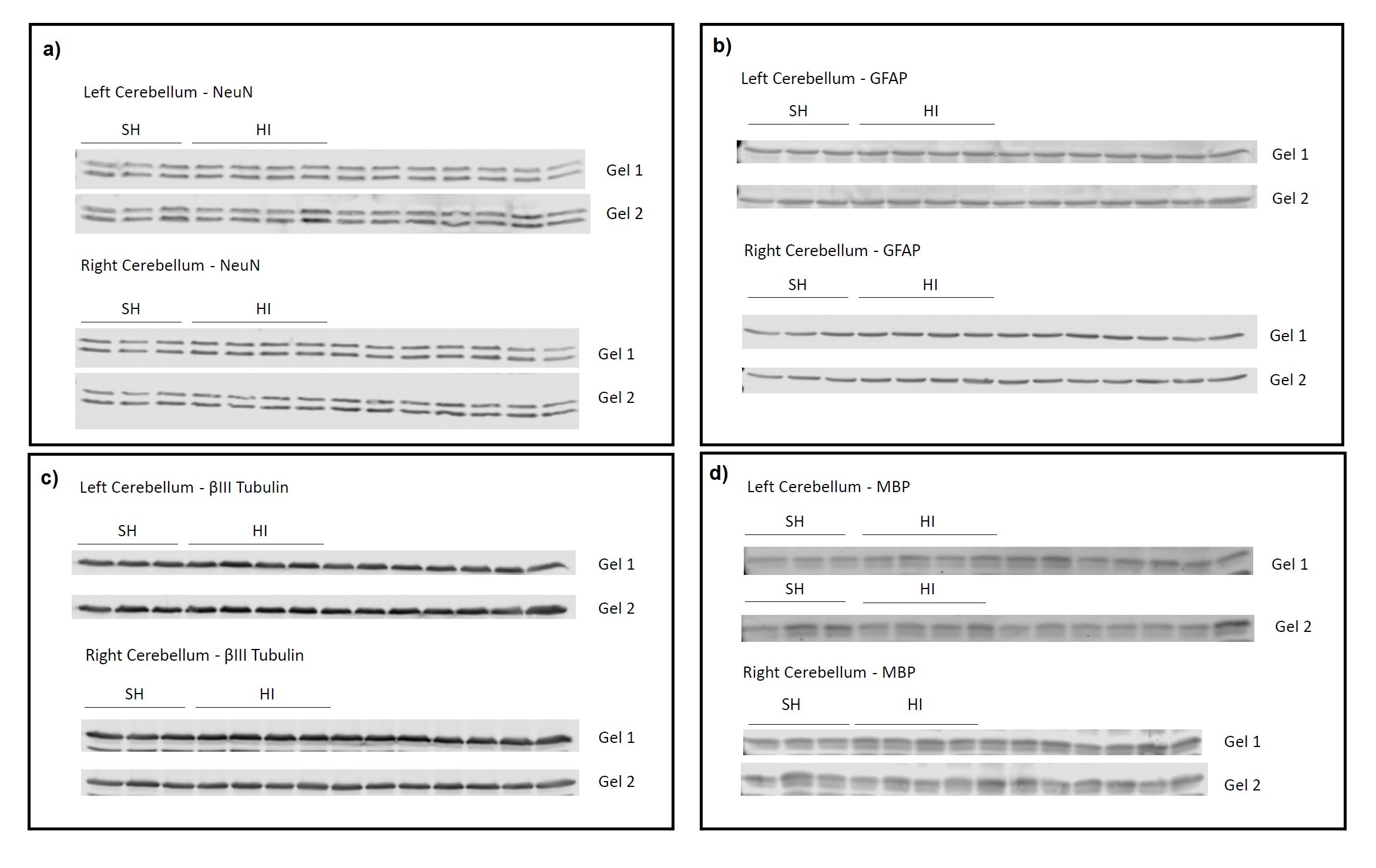

Supplement: FIGURE S1 — Original western blotting runs. Note that only the 7 ladders localized in the left side of the gels were used in the study (n = 6–8 animals/group). Antibodies used in the study were NeuN (a), GFAP (b), βIII Tubulin (c), and MBP (d). [file Image_1.tif]

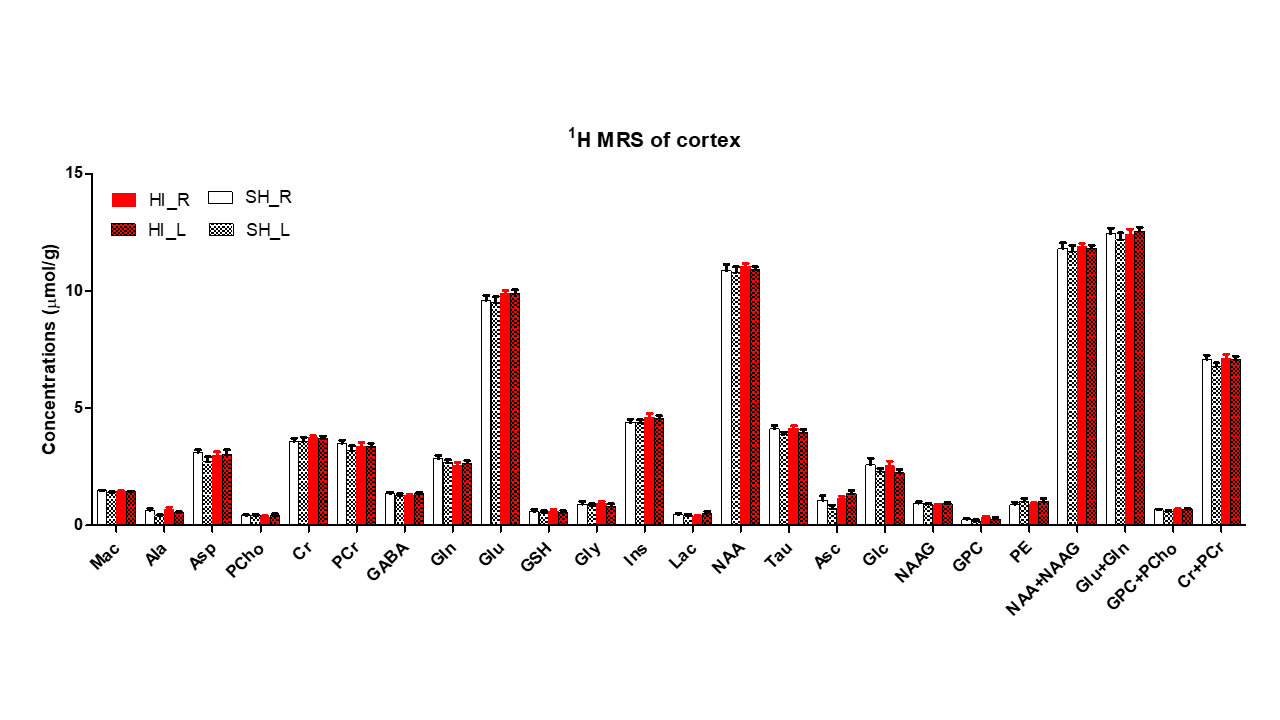

Supplement: FIGURE S2 — Metabolite concentrations in the brain cortex (R: right and L: left) after neonatal HI compared to the sham-operated animals (SH). Metabolite abbreviations were presented in the Methods section. No substantial differences were observed. [file Image_2.tif]
