# Supplementary material for: Mild Neonatal Brain Hypoxia-Ischemia in Very Immature Rats Causes Long-Term Behavioral and Cerebellar Abnormalities at Adulthood
Source: Front Physiol. 2019 Jun 5;10:634. doi: 10.3389/fphys.2019.00634 (PMC6560160; doi:10.3389/fphys.2019.00634)
Supplement: TABLE S1 — Antibodies used in the study. [file Table_1.doc]

**Supplementary Table** **1.** Antibodies used in the study

| **GFAP** | Sigma | G6171 | mouse | 55 kDa |
| --- | --- | --- | --- | --- |
| **NeuN** | Milllipore | MAB377 | mouse | 46/48 kDa |
| **Tubulin** | Abcam | ab18207 | rabbit | 50 kDa |
| **MBP** | Abcam | ab40390 | rabbit | 18 KDa |
