# Supplementary material for: Mild Neonatal Brain Hypoxia-Ischemia in Very Immature Rats Causes Long-Term Behavioral and Cerebellar Abnormalities at Adulthood
Source: Front Physiol. 2019 Jun 5;10:634. doi: 10.3389/fphys.2019.00634 (PMC6560160; doi:10.3389/fphys.2019.00634)

Image ID: 0000162_01
Acquire Time: 9 août 2017 00:00:00

Acquisition Information

#	Image ID	Acquire Time	Channels	Resolution	Intensities	Image Name	Comment	Image Modifications
1	0000162_01	9 août 2017 00:00:00	700 800	169um	5,0 5,0	WB8.8.17LCerebGFAP		

Image Display Values

Channel	Color	Minimum	Maximum	K
700	Gray Scale (Black on White)	468	24900	1

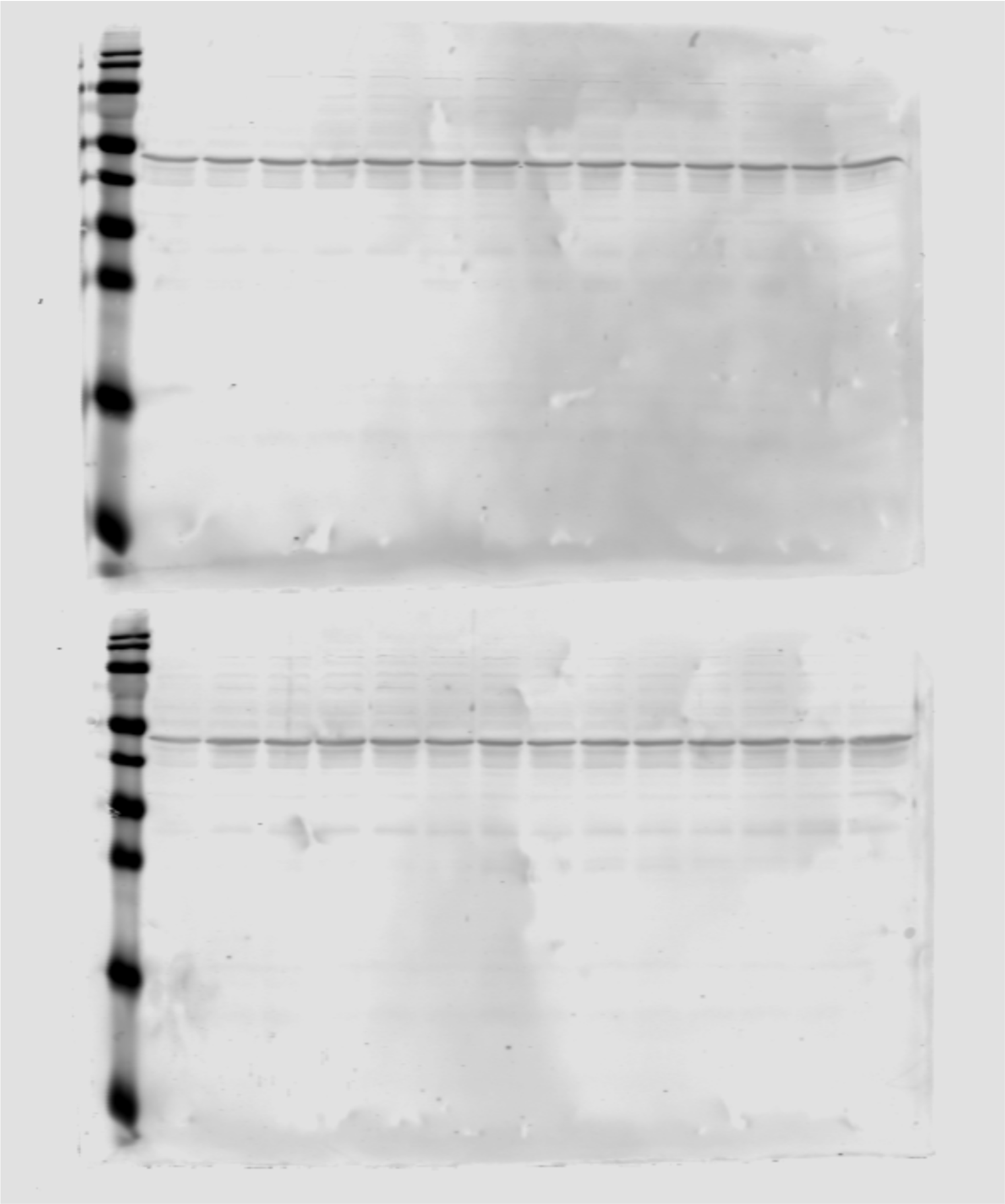

Image ID: 0000159_01
Acquire Time: 8 août 2017 00:00:00

Acquisition Information

#	Image ID	Acquire Time	Channels	Resolution	Intensities	Image Name	Comment	Image Modifications
1	0000159_01	8 août 2017 00:00:00	700	169um	5,0 5,0	WB7.8.17RCerebGFAP		

Image Display Values

Channel	Color	Minimum	Maximum	K
700	Gray Scale (Black on White)	1940	7780	0

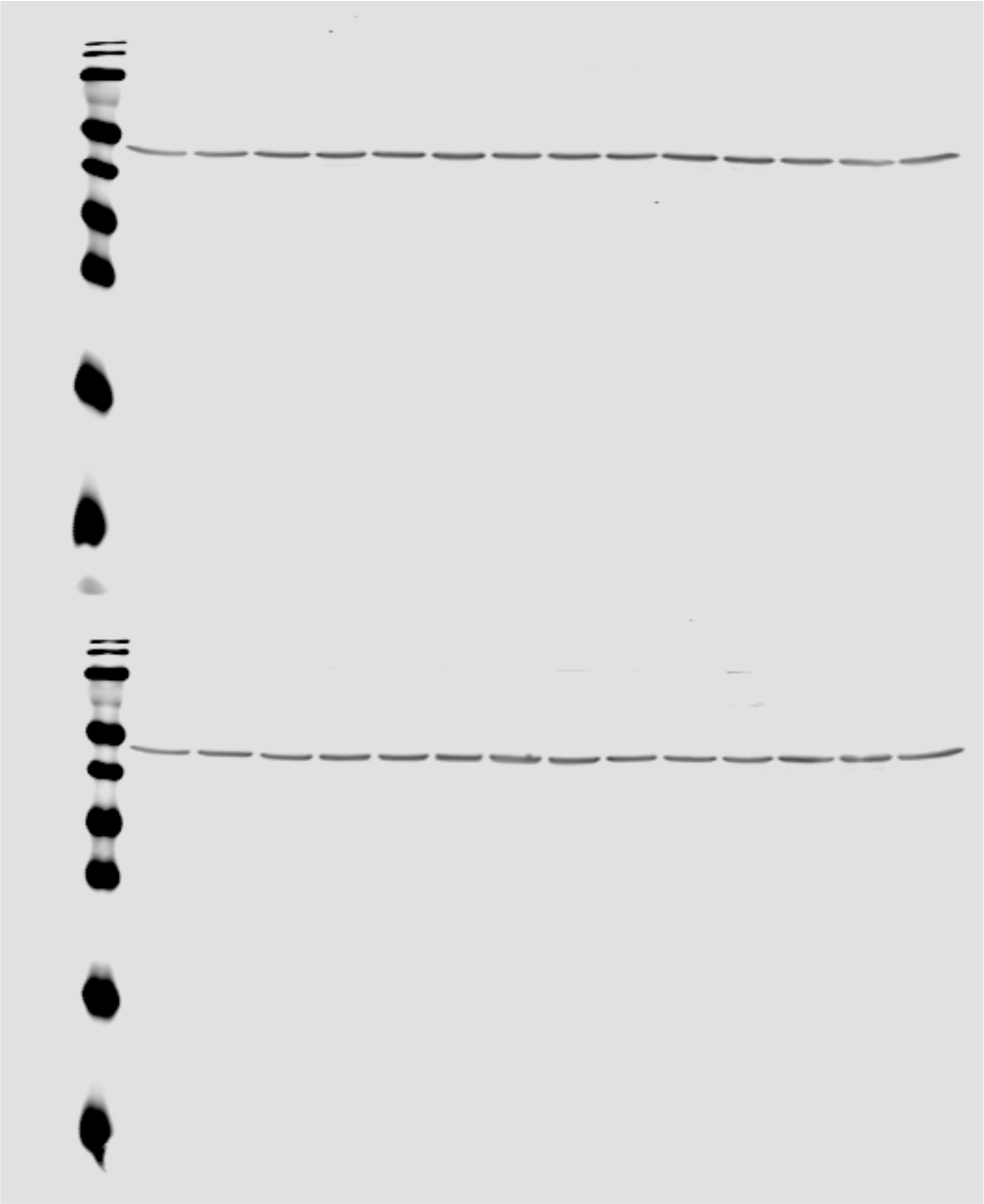

Image ID: 0000163_01
Acquire Time: 10 août 2017 00:00:00

Acquisition Information

#	Image ID	Acquire Time	Channels	Resolution	Intensities	Image Name	Comment
1	0000163_01	10 août 2017 00:00:00	700 800	169um	5,0 5,0	WB8.8.17LCerebMBPonGFAP	

Image Display Values

Channel	Color	Minimum	Maximum	K
700	Gray Scale (Black on White)	25,9	2920	0
800	Gray Scale (Black on White)	48,0	369	1

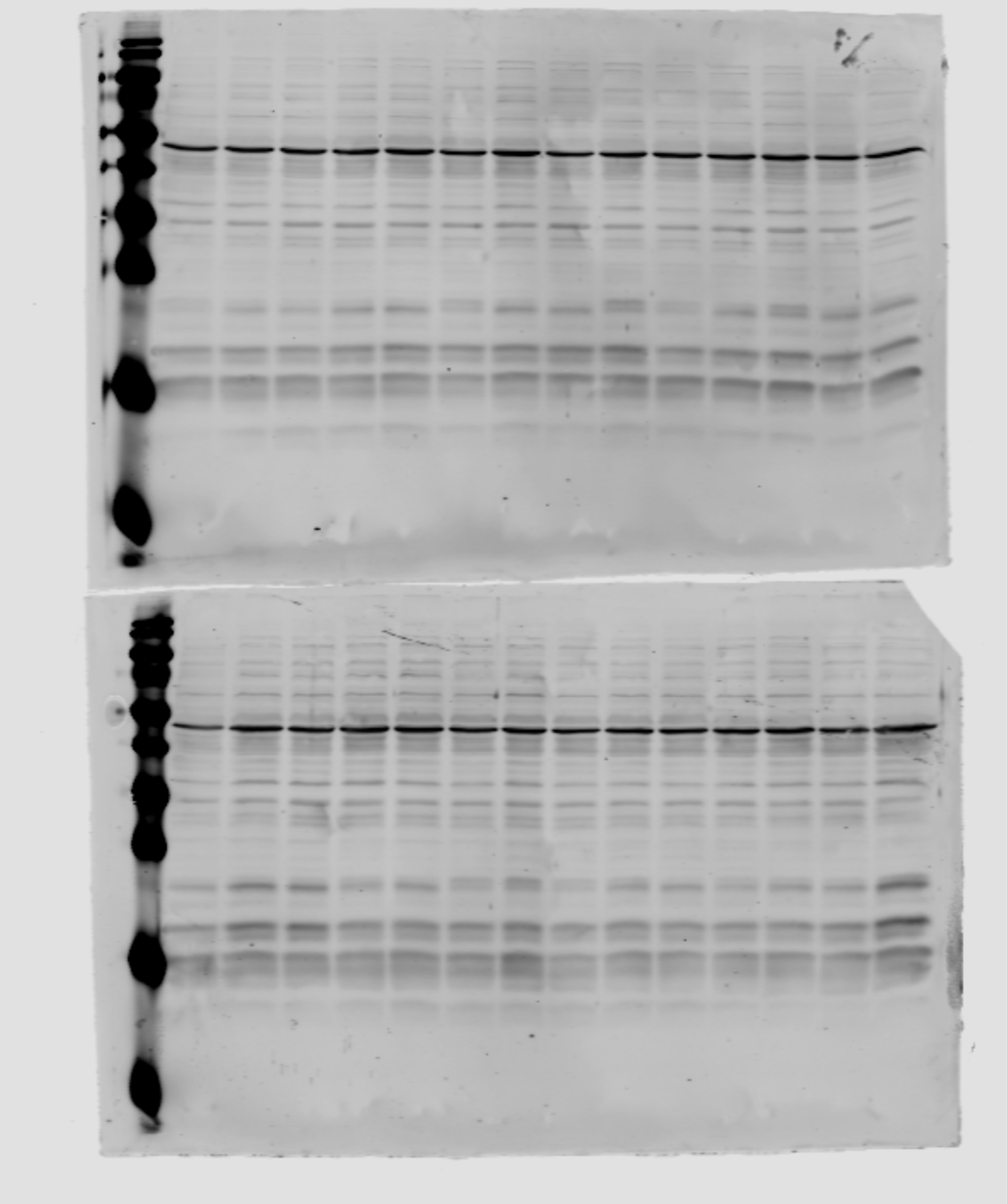

Acquisition Information

#	Image ID	Acquire Time	Channels	Resolution	Intensities	Image Name	Comment
1	0000160_01	9 août 2017 00:00:00	700 800	169um	5,0 5,0	WB7.8.17RCerebMBPonGFAP	

Image Display Values

Channel	Color	Minimum	Maximum	K
700	Gray Scale (Black on White)	915	6590	1
800	Gray Scale (Black on White)	162	1330	0

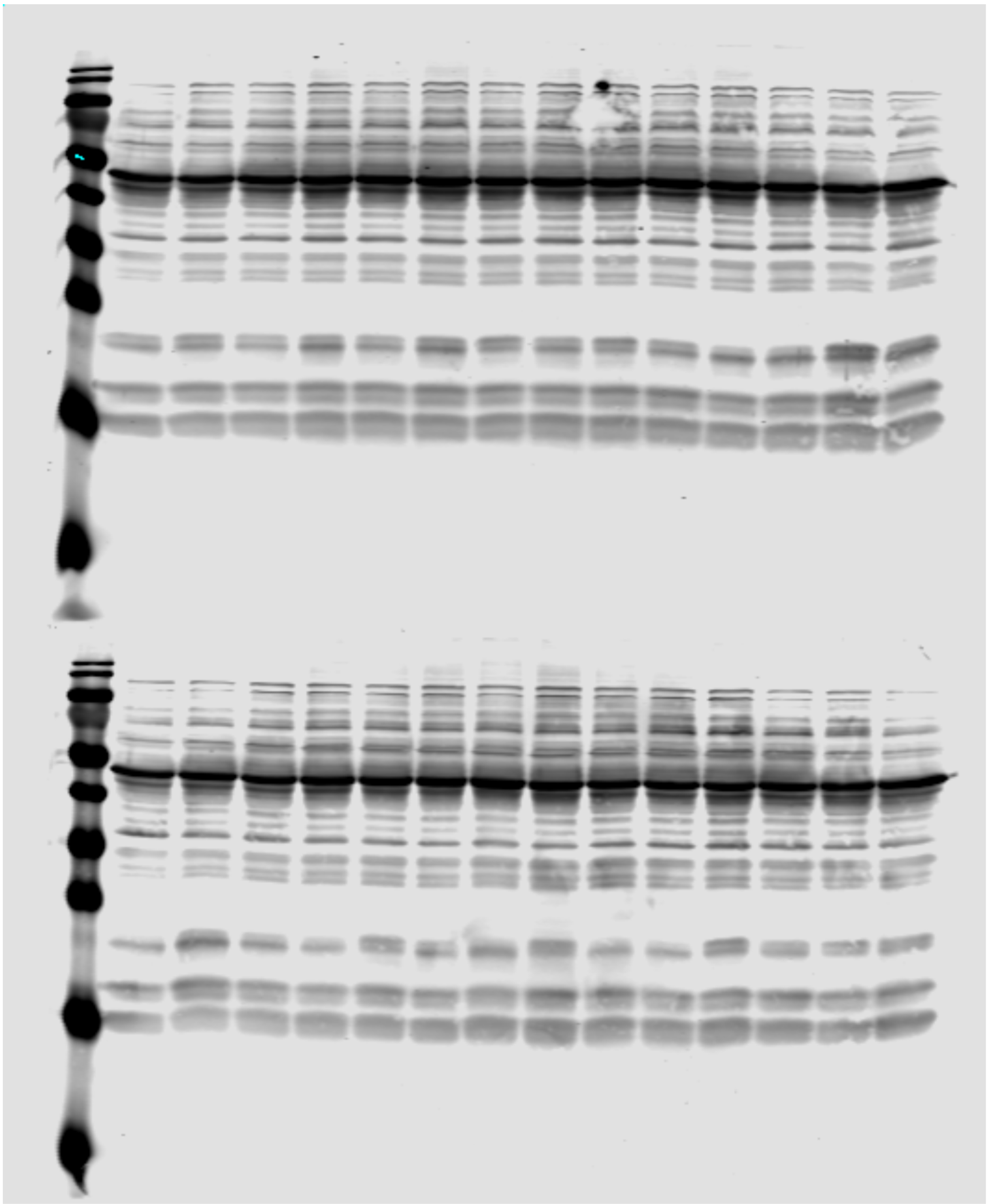

Image ID: 0000229_01
Acquire Time: 16 août 2017 00:00:00

Acquisition Information

#	Image ID	Acquire Time	Channels	Resolution	Intensities	Image Name	Comment	Image Modifications
1	0000229_01	16 août 2017 00:00:00	700 800	169um	5,0 5,0	WB15.8.17LCerebNeuN		

Image Display Values

Channel	Color	Minimum	Maximum	K
700	Gray Scale (Black on White)	844	8440	0

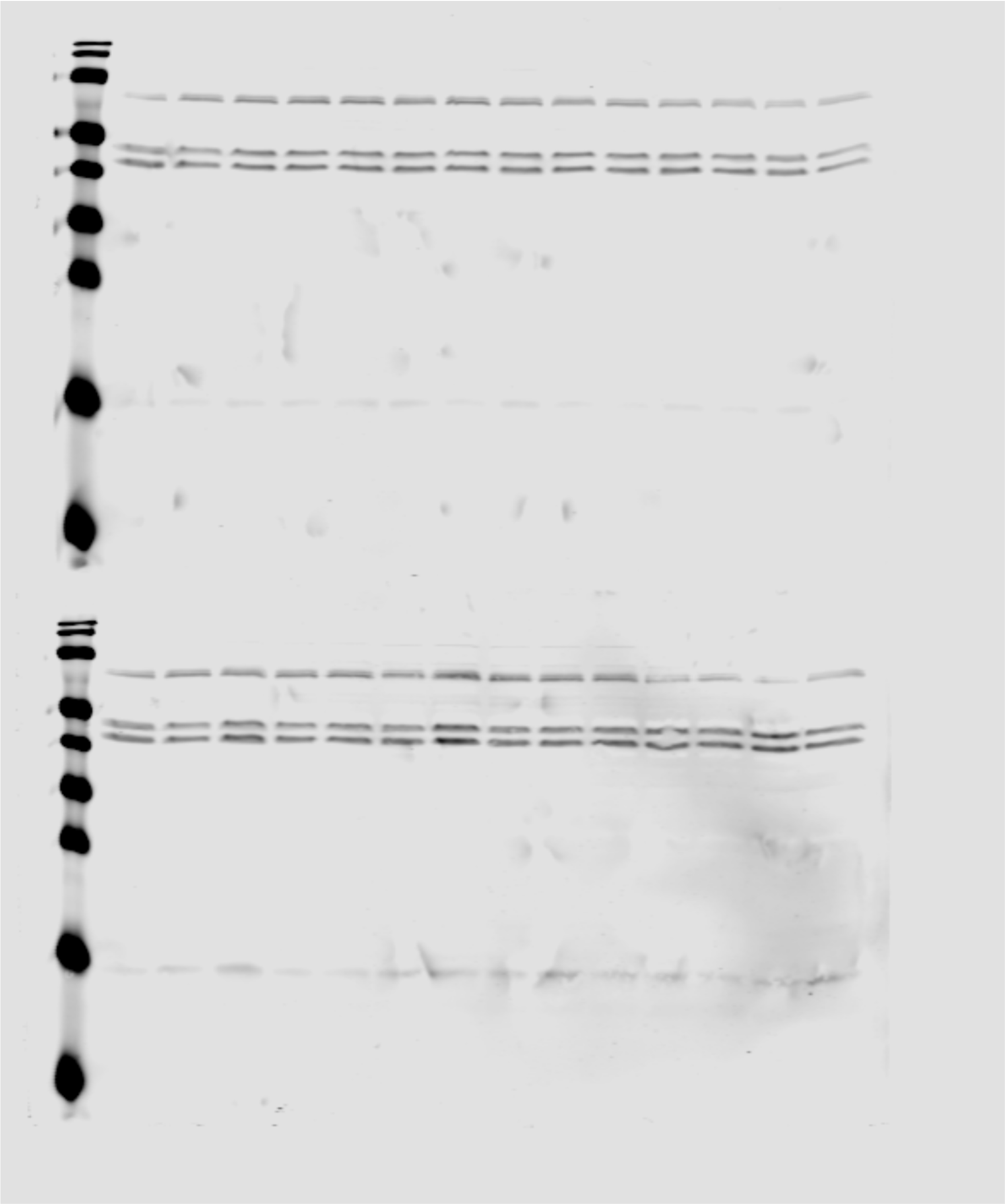

LI-COR®

Image ID: 0000158_01
Acquire Time: 15 août 2017 00:00:00

Acquisition Information

#	Image ID	Acquire Time	Channels	Resolution	Intensities	Image Name	Comment	Image Modifications
1	0000158_01	15 août 2017 00:00:00	700 800	169um	5,0 5,0	WB14.8.17RCerebNeuN		

Image Display Values

Channel	Color	Minimum	Maximum	K
700	Gray Scale (Black on White)	904	17700	1

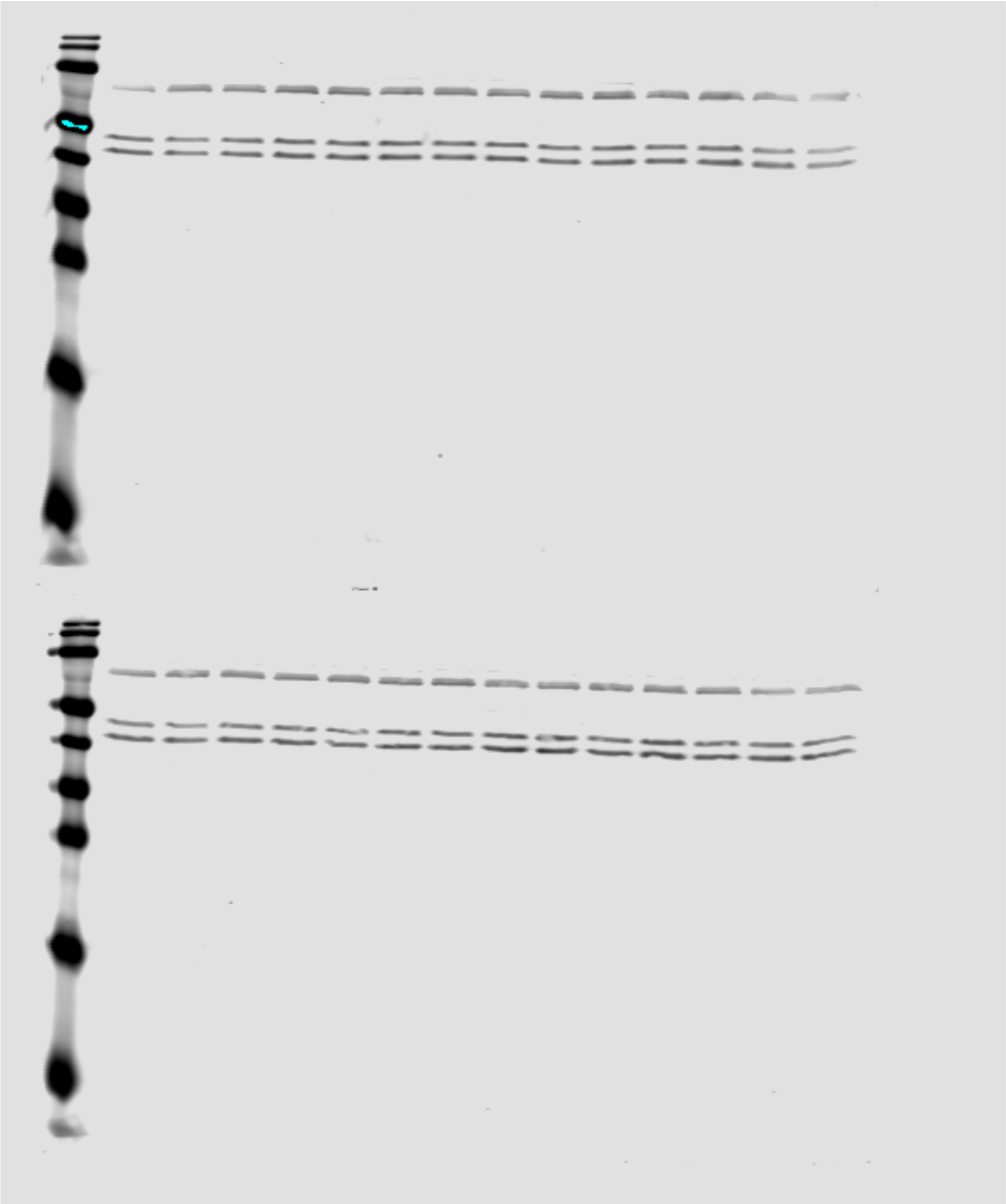

Image ID: 0000165_01
Acquire Time: 11 août 2017 00:00:00

Acquisition Information

#	Image ID	Acquire Time	Channels	Resolution	Intensities	Image Name	Comment
1	0000165_01	11 août 2017 00:00:00	700 800	169um	4,0 4,0	WB8.8.17LCerebTubulinonMBPonGFAP	

Image Display Values

Channel	Color	Minimum	Maximum	K
800	Gray Scale (Black on White)	27,0	6180	0

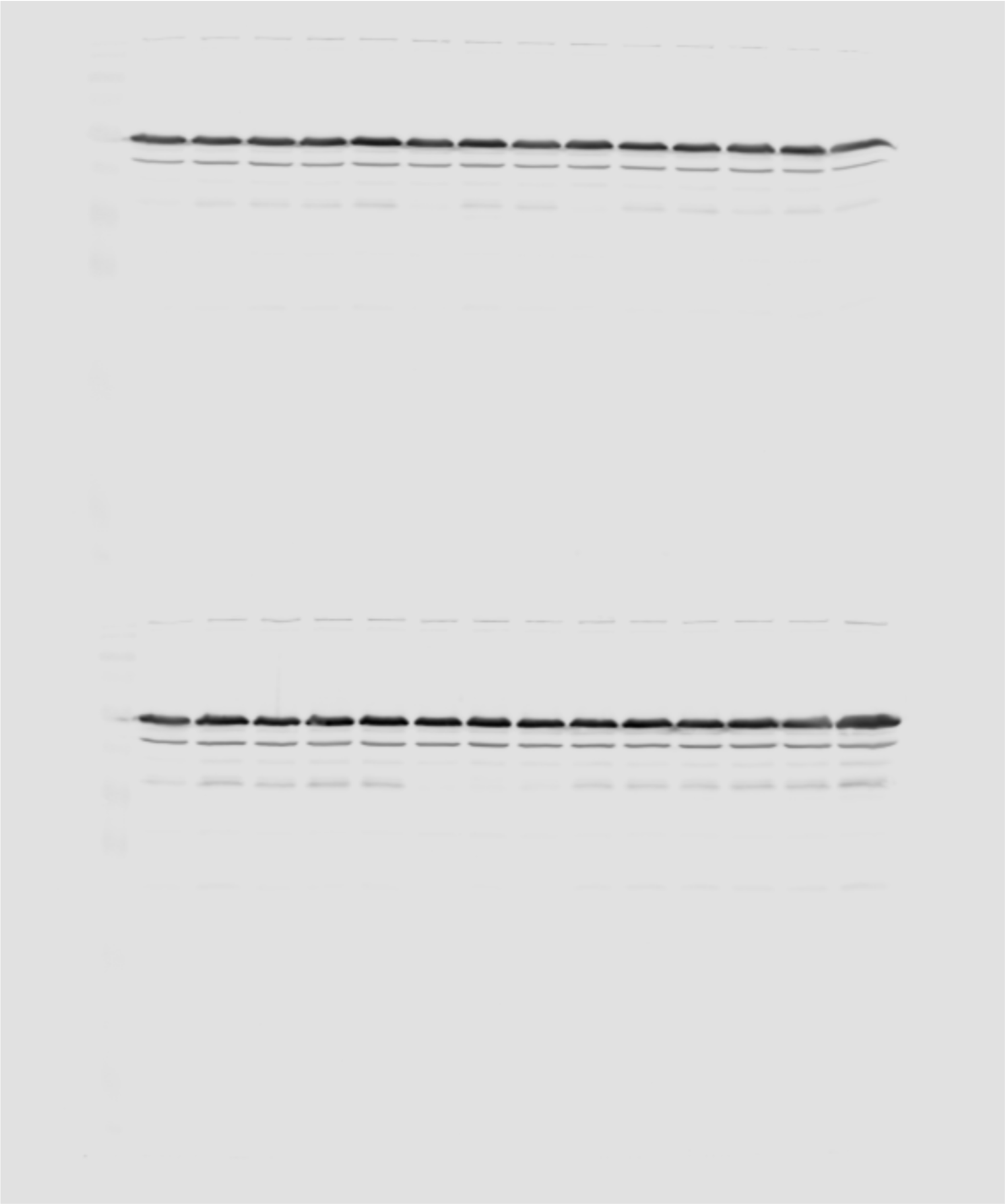

Image ID: 0000161_01
Acquire Time: 10 août 2017 00:00:00

Acquisition Information

#	Image ID	Acquire Time	Channels	Resolution	Intensities	Image Name	Comment
1	0000161_01	10 août 2017 00:00:00	700 800	169um	2,0 2,5	WB7.8.17RCerebTubulinonMBPonGFAP	

Image Display Values

Channel	Color	Minimum	Maximum	K
800	Gray Scale (Black on White)	14,0	7840	0

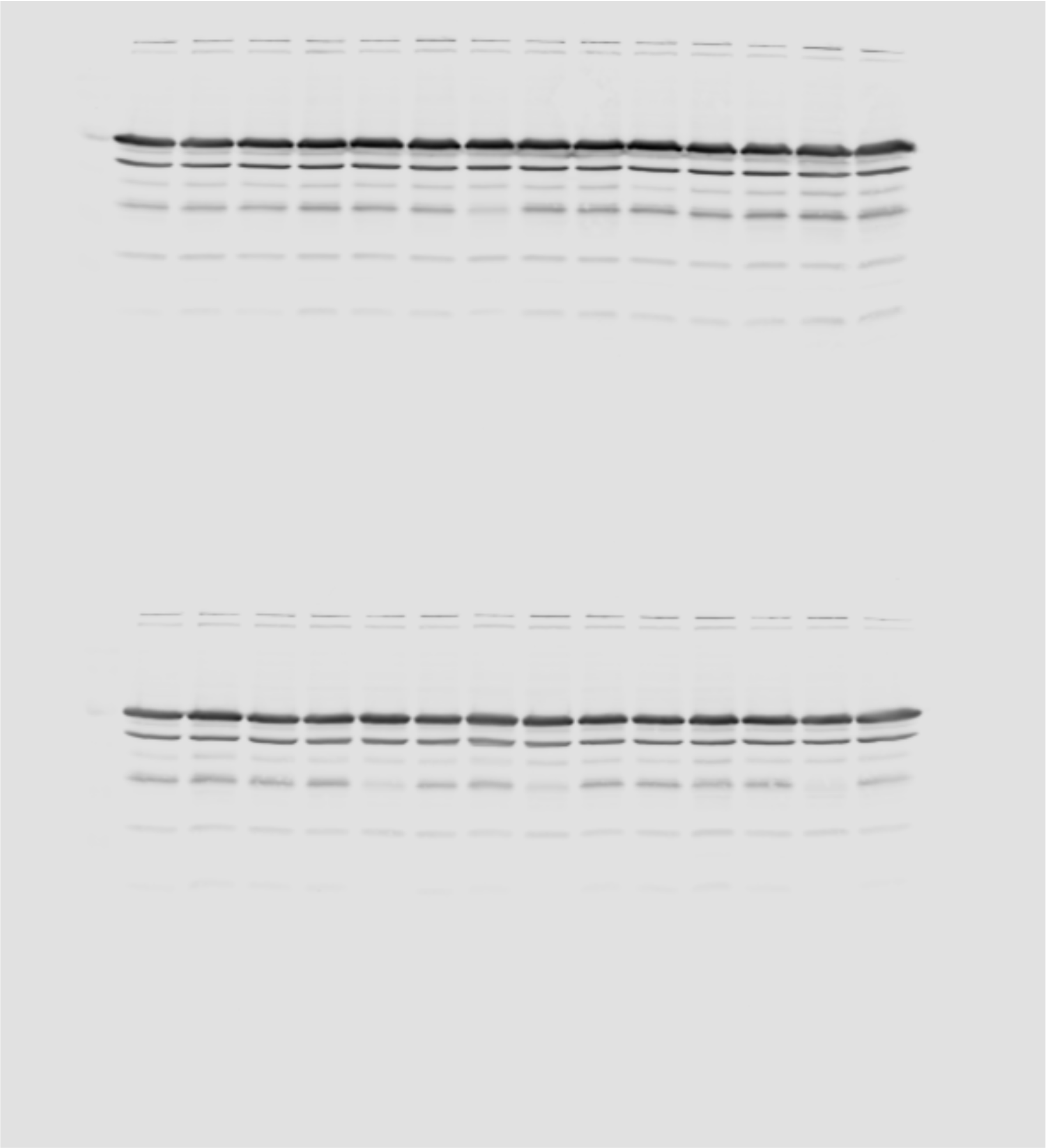

Supplement: DATA SHEET S1 — The raw data of typical western blotting runs. [file Data_Sheet_1.PDF]
